# Vitamin D, Fibroblast Growth Factor 23 and Incident Cognitive Impairment: Findings from the REGARDS Study

**DOI:** 10.1371/journal.pone.0165671

**Published:** 2016-11-03

**Authors:** Bhupesh Panwar, Suzanne E. Judd, Virginia J. Howard, Nancy S. Jenny, Virginia G. Wadley, Orlando M. Gutiérrez

**Affiliations:** 1 Department of Medicine, University of Alabama at Birmingham, Birmingham, AL, United States of America; 2 Department of Biostatistics, University of Alabama at Birmingham, Birmingham, AL, United States of America; 3 Department of Epidemiology, University of Alabama at Birmingham, Birmingham, AL, United States of America; 4 Department of Pathology, University of Vermont College of Medicine, Burlington, VT, United States of America; Medizinische Universitat Innsbruck, AUSTRIA

## Abstract

Vitamin D protects against cognitive decline in animals but evidence in humans has been inconsistent. Fibroblast growth factor 23 (FGF23) is a hormone that inhibits vitamin D activation yet few studies examined whether FGF23 is associated with cognitive impairment. The objective of this study was to examine associations of 25(OH)D and FGF23 with incident cognitive impairment in the Reasons for Geographic and Racial Differences in Stroke (REGARDS) study, a cohort of black and white adults ≥45 years old. FGF23 and 25(OH)D were measured in 474 incident impairment cases and 561 controls. In multivariable-adjusted models, there were no significant associations of FGF23 with incident cognitive impairment. In analyses using clinically-relevant categories of 25(OH)D (< 20 ng/ml, 20–29.9 ng/ml, ≥30 ng/ml), there was no statistically significant association of lower 25(OH)D concentrations with odds of incident cognitive impairment in models adjusted for demographic, clinical, and laboratory variables and season of blood draw (tertile 1 [≥30 ng/ml] reference; tertile 2 [20–29.9 ng/ml], odds ratio [OR] 0.96, 95%CI 0.67, 1.38; tertile 3 [<20 ng/ml] OR 1.26, 95%CI 0.83, 1.91). When 25(OH)D was modeled as race-specific tertiles, there were no significant associations of 25(OH)D with incident cognitive impairment in whites, whereas lower 25(OH)D was associated with higher odds in blacks (tertile 1 [>23 ng/ml] reference; tertile 2 [15–23 ng/ml], OR 2.96, 95%CI 1.48,5.94; tertile 3 [<15 ng/ml] OR 2.40, 95%CI 1.07,5.40) in the fully adjusted model. In this cohort of older adults, lower race-specific tertiles of 25(OH)D were associated with higher incidence of cognitive impairment in black individuals but not white individuals. These data suggest that treating low 25(OH)D may be a novel strategy for addressing racial disparities in neurocognitive outcomes.

## Introduction

Cognitive impairment is a debilitating condition in older adults. Because the costs associated with the care of individuals with cognitive impairment are high and growing in parallel with the aging population,[1] identification of potentially modifiable risk factors for the development or progression of cognitive impairment is a high priority.

Vitamin D is a hormone that has important neuroprotective effects. Vitamin D receptors (VDRs) are expressed abundantly in the central nervous system (CNS) and neurons have the ability to synthesize 1,25-dihydroxyvitamin D, the activated form of vitamin D.[2–4] Animal studies have shown that vitamin D supplementation attenuates the development of cognitive decline in aging rats.[5–12] The importance of vitamin D for neurocognitive outcomes in humans is less clear. Whereas some prospective studies showed an inverse association of 25-hydroxyvitamin D (25(OH)D) concentrations with incident cognitive impairment,[13–18] others showed no significant associations when accounting for traditional risk factors.[19–21] Most prior studies were relatively small in sample size and/or lacked race or sex heterogeneity, potentially explaining inconsistencies in their results. Prior studies were also limited by a lack of data on key hormones involved in the regulation of vitamin D activity, such as fibroblast growth factor 23 (FGF23). This is important in that FGF23 strongly inhibits the activation of vitamin D, which is essential for up-regulating vitamin D-dependent signal transduction pathways shown to be neuroprotective. Accordingly, we examined the association of circulating 25(OH)D and FGF23 with incident cognitive impairment in the Reasons for Geographic and Racial Differences in Stroke (REGARDS) Study, a large cohort of black and white adults ≥45 years old.

## Methods

The Reasons for Geographic and Racial Differences in Stroke (REGARDS) study is a population-based investigation of stroke incidence in United States (US) adults. Details of the study design have been reported elsewhere.[22] Briefly, the REGARDS study recruited participants between 2003 and 2007 and has been continuously following participants since baseline. REGARDS enrolled a large national study of participants aged 45 years and older and was designed to be evenly balanced in terms of race (black and white), geography (Southeastern US and the rest of the nation), and sex. Potential participants were initially mailed a letter inviting them to participate followed by a baseline telephone interview lasting approximately 45 minutes. Following initial verbal consent during the telephone interview, a trained health professional went to the participant’s home to collect blood and urine samples, and obtain blood pressure measurements, an electrocardiogram (ECG), other key study variables, and written consent. Blood was stored and analyzed at the central lab at the University of Vermont and ECGs were centrally read at Wake Forest University. The final study sample included 30,239 participants (42% black and 55% female). The REGARDS study protocol was approved by the Institutional Review Boards governing research in human subjects at the participating centers (the University of Alabama at Birmingham Institutional Review Board for Human Use; the University of Vermont Institutional Review Board; the Wake Forest University Institutional Review Board) and all participants provided written informed consent.

### Primary Exposure

The exposures of interest were FGF23 and 25(OH)D concentrations measured in baseline blood samples. FGF23 was measured using a second generation, C-terminal enzyme linked immunosorbent assay (Immutopics, Santa Clara, CA) with coefficients of variation <10%. 25(OH)D was measured using a commercially-available ELISA (Immunodetection Systems, Fountain Hills, AZ) with appropriate high and low value controls. The assay range was 5–150 ng/ml. Intra-assay CVs were 8.82–12.49%.

### Outcome of Interest

The outcome of interest was incident cognitive impairment. Cases of incident cognitive impairment were defined based upon 4 cognitive tests administered during the phone interviews: a baseline 6-item screener and a follow-up 3-test measure including an animal fluency test, a word list learning test, and a word list recall.[23, 24] The 6-item screener is a test of global cognitive function that assesses recall of a 3-item word list and temporal orientation (year, month, day of the week), with scores ranging from 0 to 6.[23] Animal fluency is a verbal fluency test scored as the number of animals that a participant can name in 60 seconds, and word list learning and word list recall measure ability to learn and recall a 10-item list.[24, 25] We excluded participants with baseline cognitive impairment (6-item screener score ≤ 4),[23] baseline self-reported stroke, insufficient cognitive testing, or anomalous data. The remaining eligible participants (n = 17,630) were defined as developing incident cognitive impairment if they had a score ≥ 1.5 standard deviations (SD) below age-, race-, sex-, and education-adjusted predicted scores on 2 or 3 of the 3 other tests during follow-up.[26] These cut-points were in part chosen to ensure that we captured sufficient numbers of individuals who developed substantial cognitive impairment.

### Study Design

We used a case-control study design. A total of 495 cases of incident cognitive impairment were identified using the criteria described above. The 587 unmatched controls were participants from a 1100-person stratified random sample of the REGARDS cohort who met the eligibility criteria applied to cases. This cohort random sample was selected using stratified sampling to ensure sufficient representation of high-risk groups. All participants with at least one follow-up contact (n = 29,653) were categorized into 20 strata based on age (45–54, 55–64, 65–74, 75–84, ≥85 years), race (black or white), and sex (male or female).[27] In each stratum, participants were randomly selected to fulfill the desired distribution: 50% black, 50% white, 50% female, 50% male, 20% age 45–54, 20% age 55–64, 25% age 65–74, 25% age 75–84, and 10% age ≥85.

### Covariates of Interest

Age, race, sex, education, smoking history, and annual household income were determined by self-report. Systolic and diastolic blood pressure were defined as the average of two seated measures taken after a 5 minute rest. Body mass index (BMI) was calculated as weight in kilograms divided by height in meters squared. Waist circumference (in centimeters) was measured using a tape measure positioned midway between the lowest rib and the iliac crest with the participant standing. History of coronary heart disease (CHD) was defined as having any of the following: evidence of myocardial infarction on the baseline ECG, self-report of a prior history of a cardiac procedure (coronary artery bypass surgery or percutaneous coronary intervention), or self-reported history of myocardial infarction. Diabetes was defined as self-reported use of insulin or oral hypoglycemic agents, fasting blood glucose concentration of 6.9 mmol/L or higher, or a non-fasting blood glucose concentration of 11.0 mmol/L or higher. The 4-item Centers for Epidemiologic Studies of Depression (CESD-4) scale was used to assess depressive symptoms [28]. The scale assesses how many days in the prior week participants felt depressed, felt lonely, had crying spells, and felt sad, with response options including: < 1 day (0 points), 1–2 days (1 point), 3–4 days (2 points), and 5–7 days (3 points). Each item is scored individually and then all items are summed, with the total score ranging from 0 to 12 points. Participants with a CESD-4 score ≥4 points were categorized as having depressive symptoms. Phosphorus and calcium concentrations were measured in baseline blood samples using standard assays. Serum intact parathyroid hormone concentrations (PTH) were measured using a commercially available ELISA (Roche Elecsys 2010, Roche Diagnostics, Indianapolis, IN). Estimated glomerular filtration rate (eGFR) was determined from serum creatinine measurements using the Chronic Kidney Disease (CKD) Epidemiology Collaboration equation.[29] Urine albumin measured by the BNII ProSpec nephelometer (Siemens AG) and urine creatinine measured by the rate Jaffé method (Roche/Hitachi, Basel, Switzerland) were used to calculate urine albumin to creatinine ratio (ACR). Chronic kidney disease (CKD) was defined as an eGFR <60 ml/min/1.73m2 or an ACR >30 mg/g.

### Statistical Analysis

Descriptive statistics were used to compare characteristics in participants with cognitive impairment vs. controls. To account for the stratified sampling design of controls from the random cohort, all analyses were weighted by the inverse of the random cohort sampling fraction to weight each control back to the original cohort.[30] Odds ratios (OR) of incident cognitive impairment as a function of baseline FGF23 or 25(OH)D were examined with weighted logistic regression models. Model 1 was unadjusted. Model 2 adjusted for geographic region of residence, income, diabetes status, CHD, smoking status (current vs. never or past) and depressive symptoms (yes or no). Model 3 adjusted for variables in Model 2 plus eGFR, log-transformed ACR, and other markers of mineral metabolism (phosphorus, calcium and PTH concentrations). In models with FGF23 as the primary predictor variable, FGF23 was analyzed in quartiles, with the lowest quartile serving as the referent group, and on a continuous scale. FGF23 concentrations were not normally distributed—therefore, in analyses modeling FGF23 as a continuous variable, FGF23 was evaluated after log base 2 transformation (interpreted as “per doubling” of FGF23). In models with 25(OH)D as the primary predictor variable, 25(OH)D was analyzed in clinically-relevant categories (<20 ng/ml, 20–29.9 ng/ml, ≥30 ng/ml), with the highest category (≥30 ng/ml) serving as the referent group, and on a continuous scale. Given wide variability in the distribution of 25(OH)D concentrations by race [31–34], in pre-specified analyses, we also examined the same associations using race-specific tertiles of 25(OH)D in the study sample overall and stratified by race. We examined for effect modification by CKD and race by testing the statistical significance (*P*<0.10) of a multiplicative interaction term in the model. A two-tailed *P* value <0.05 was considered statistically significant except for the models examining interaction. All analyses were conducted using SAS software version 9.4 (SAS Institute, Cary, NC).

## Results

After excluding 47 participants who had missing values for either 25(OH)D or FGF23 (21 cases and 26 controls), a total of 474 cases with a mean follow-up of 3.5 ± 1.8 years and 561 controls were included in the final analyzed sample. Table 1 shows the baseline characteristics of cases as compared to the controls. Cases were more likely to live in the US stroke belt, have lower income, have diabetes, CHD, and CKD, be current smokers, and have lower 25(OH)D concentrations and higher FGF23 concentrations than controls.

**Table 1:** Baseline characteristics in cases as compared to controls. Values are depicted as mean (95% confidence interval), frequencies, or median [interquartile range].

	Case	Control	*P*-value
	n = 474	weighted n = 16,373	
Age	64.6 (63.9, 65.3)	64.2 (63.7, 64.6)	0.39
Black race (%)	33	36	0.28
Body mass index (kg/m^2^)	29.9 (29.5, 30.5)	29.2 (28.7, 29.7)	0.05
Male sex (%)	42	43	0.72
Region of residence (%)			<0.001
Non-belt	35	48	
Buckle	22	20	
Belt	44	32	
Income < $20,000 per year (%)	28	14	<0.001
Less than a high school diploma (%)	8	7	0.63
Co-morbidities			
Diabetes (%)	28	17	<0.001
Hypertension (%)	61	55	0.06
Coronary heart disease (%)	20	14	0.005
Current smoking (%)	17	12	0.04
Physical activity, none (%)	38	32	0.07
Chronic kidney disease (%)	26	17	<0.001
Laboratory measures			
Calcium (mg/dL)	9.16 (9.10, 9.22)	9.25 (9.20, 9.30)	0.08
Phosphorus (mg/dL)	3.48 (3.44, 3.52)	3.52 (3.47, 3.56)	0.24
Parathyroid hormone (pg/ml)	47.1 (44.4, 49.8)	43.4 (41.8, 45.0)	0.15
25-hydroxyvitamin D (ng/ml)	25.8 (24.8, 26.7)	26.8 (25.9, 27.6)	0.03
Fibroblast growth factor 23 (RU/ml)	74.3 [56.2, 112.4]	67.9 [51.9, 97.0]	0.03

### Associations of baseline FGF23 concentrations with incident cognitive impairment

Odds ratios of developing cognitive impairment by baseline FGF23 concentrations are shown in Table 2. In the unadjusted model, when compared to the lowest quartile of FGF23 (FGF23 <53 RU/ml), the highest quartile of FGF23 (FGF23 >100 RU/ml) had a 73% higher odds of incident cognitive impairment (OR 1.73, 95% confidence interval [CI] 1.21,2.47). Similarly, when FGF23 was modeled as a continuous variable, in the unadjusted model, higher FGF23 concentrations were associated with higher odds of developing cognitive impairment (OR per doubling of FGF23 1.12, 95%CI 1.03,1.33). These associations were attenuated and no longer statistically significant after adjustment for demographic and clinical variables and after further adjustment for laboratory variables. Neither presence of CKD nor race modified these associations (*P*_interaction_>0.1 for both).

**Table 2:** Odds ratio (95% confidence interval) of incident cognitive impairment according to baseline fibroblast growth factor 23 concentrations.

	FGF23 Quartile 1(<53 RU/ml)	FGF23 Quartile 2(53–69.9 RU/ml)	FGF23 Quartile 3(70–100 RU/ml)	FGF23 Quartile 4(>100 RU/ml)	Per doubling of FGF23
Events	94	118	115	147	474
Model 1	ref	1.27 (0.89, 1.83)	1.31 (0.91, 1.87)	1.73 (1.21, 2.47)	1.12 (1.03, 1.33)
Model 2	ref	1.35 (0.89, 2.05)	1.13 (0.73, 1.74)	1.19 (0.76, 1.84)	1.01 (0.87, 1.18)
Model 3	ref	1.23 (0.80, 1.89)	1.08 (0.68, 1.72)	1.04 (0.63, 1.72)	0.99 (0.81, 1.23)

Model 1 is unadjusted; Model 2 is adjusted for geographic region of residence, annual income, diabetes status, history of coronary heart disease, current smoking and depressive symptoms; Model 3 is adjusted for variables in model 2 plus estimated glomerular filtration rate, log-transformed albumin to creatinine ratio, phosphorus, calcium, and parathyroid hormone.

### Associations of baseline 25(OH)D concentrations with incident cognitive impairment

Table 3 depicts the odds ratios of developing cognitive impairment by baseline 25(OH)D concentrations. The lowest tertile of 25(OH)D (< 20 ng/ml) was associated with a significantly higher odds of developing cognitive impairment when compared to the highest tertile (≥ 30 ng/ml) in the unadjusted model (OR 1.55 95%CI, 1.13,2.13). This association was attenuated and no longer statistically significant after adjustment for sociodemographic, clinical and laboratory variables and season of blood draw. When examined on a continuous scale, there was no association of 25(OH)D with odds of developing cognitive impairment in either unadjusted or adjusted models. Neither presence of CKD nor race modified these associations (*P*_interaction_>0.1 for both).

**Table 3:** Odds ratio (95% confidence interval) of incident cognitive impairment according to categories of baseline 25-hydroxyvitamin D concentrations.

	25(OH)D Category 1(≥ 30 ng/ml)	25(OH)D Category 2(20–29.9 ng/ml)	25(OH)D Category 3(< 20 ng/ml)	Per 1 ng/ml change in25(OH)D
Events	148	159	167	474
Model 1	ref	0.91 (0.68, 1.23)	1.55 (1.13, 2.13)	0.99 (0.98, 1.00)
Model 2	ref	0.91 (0.65, 1.28)	1.26 (0.97, 1.83)	1.00 (0.99, 1.02)
Model 3	ref	0.96 (0.67, 1.38)	1.26 (0.83, 1.91)	1.00 (0.99, 1.02)

25(OH)D, 25-hydroxyvitamin D; Model 1 is unadjusted; Model 2 is adjusted for geographic region of residence, season of blood draw, annual income, diabetes status, history of coronary heart disease, current smoking and depressive symptoms; Model 3 is adjusted for variables in model 2 plus estimated glomerular filtration rate, log-transformed albumin to creatinine ratio, phosphorus, calcium, and parathyroid hormone.

### Associations of race specific tertiles of baseline 25-hydroxyvitamin D concentrations with incident cognitive impairment

**Fig 1:**
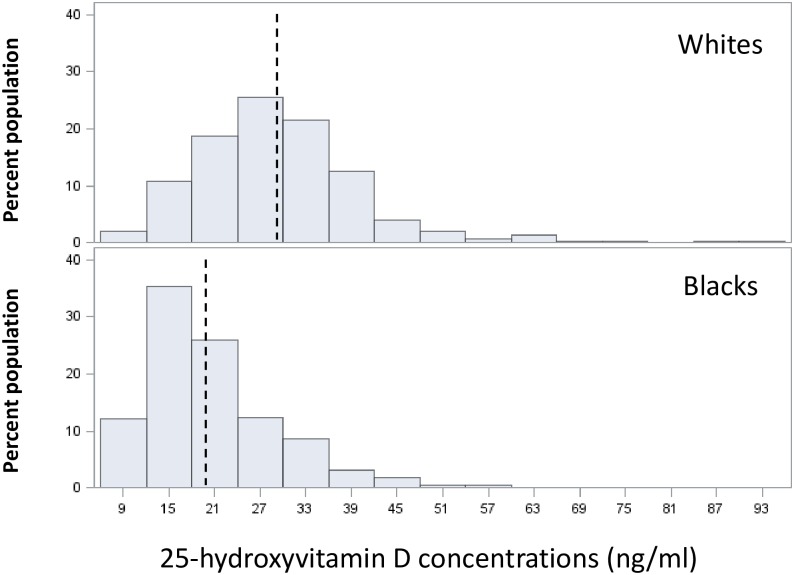
Histograms showing the distribution of 25-hydroxyvitamin D concentrations in white and black participants separately. Vertical dotted line indicates the mean 25-hydroxyvitamin D concentration in each population.

Mean 25(OH)D concentrations were lower in black as compared to white participants (Fig 1). In pre-specified analyses using race specific tertiles of 25(OH)D (Table 4), there was no statistically significant associations of lower 25(OH)D with odds of incident cognitive impairment in unadjusted or multivariable models among white participants. However, among black participants, lower 25(OH)D was associated with greater odds of developing cognitive impairment in the fully adjusted model (tertile 1 [>23 ng/ml] referent group; tertile 2 [15–23 ng/ml] OR 2.96, 95%CI 1.48,5.94; tertile 3 [<15 ng/ml] OR 2.40, 95%CI 1.07, 5.40).

**Table 4:** Odds ratio (95% confidence interval) of incident cognitive impairment according to baseline 25-hydroxyvitamin D concentrations modeled as race-specific tertiles and stratified by race.

	25(OH)D Tertile 1> 23 ng/ml for blacks> 32 ng/ml for whites	25(OH)D Tertile 215–23 ng/ml for blacks25-32 ng/ml for whites	25(OH)D Tertile 3< 15 ng/ml for blacks< 25 ng/ml for whites
**Black participants**			
Events	31	69	53
Model 1	ref	2.79 (1.64,4.76)	2.19 (1.26,3.83)
Model 2	ref	2.74 (1.48,5.07)	1.93 (0.98,3.79)
Model 3	ref	2.96 (1.48,5.94)	2.40 (1.07, 5.40)
**White participants**			
Events	105	97	119
Model 1	ref	0.94 (0.64,1.38)	1.35 (0.92,1.96)
Model 2	ref	1.01 (0.66,1.56)	1.23 (0.79,1.90)
Model 3	ref	1.12 (0.70,1.78)	1.22 (0.76, 1.96)

25(OH)D, 25-hydroxyvitamin D; Model 1 is unadjusted; Model 2 is adjusted for geographic region of residence, season of blood draw, annual income, diabetes status, history of coronary heart disease, current smoking and depressive symptoms; Model 3 is adjusted for variables in model 2 plus estimated glomerular filtration rate, log-transformed albumin to creatinine ratio, phosphorus, calcium, and parathyroid hormone

## Discussion

We found no evidence that higher FGF23 was associated with the development of cognitive impairment in community-dwelling black and white adults after accounting for potential confounders. Similarly, there was no independent association of 25(OH)D with odds of incident cognitive impairment when using standard clinical cut-points for defining 25(OH)D insufficiency or deficiency. However, when 25(OH)D categories were defined using race-specific tertiles, lower 25(OH)D concentrations were associated with higher multivariable-adjusted odds of incident cognitive impairment in black individuals but not white individuals.

Prospective studies examining the association of 25(OH)D with the development of cognitive impairment have reported inconsistent findings.[13–21] Differences in the study populations and the metrics used to define cognitive impairment in each study make it challenging to compare results across studies. Nonetheless, most prior studies were limited by being largely homogeneous in race (the vast majority were white). Only one prior study had a substantial proportion of individuals of black race and did not report any racial differences in the association of baseline 25(OH)D concentrations with cognitive decline defined by standardized neuropsychological testing performed at baseline and follow-up.[18] Thus, our study provides important context to prior studies demonstrating an association of 25(OH)D with neurocognitive disease by showing this relationship was only apparent when using race-specific tertiles of 25(OH)D. These results highlight the importance of accounting for the marked differences in the distribution of 25(OH)D by race when examining the association of 25(OH)D with neurocognitive outcomes in racially diverse populations.

The reason why lower 25(OH)D was associated with incident cognitive impairment in blacks but not whites in race-stratified analyses is not clear. However, it is possible that the much lower mean 25(OH)D concentration in blacks may play a role. Prior studies have shown that the association of low 25(OH)D with cognitive impairment was driven by individuals with very low 25(OH)D concentrations. Since black individuals were much more likely to have very low concentrations of 25(OH)D than whites in the current study, it is possible that we had greater resolution to detect an association of very low 25(OH)D with cognitive impairment in the low spectrum of 25(OH)D concentrations in blacks as compared to whites. If so, these data suggest that using race-specific tertiles may be important for detecting associations of 25(OH)D with neurocognitive outcomes in racially diverse populations.

Experimental studies have shown that vitamin D supplementation can retard the development of cognitive decline in aging rats through a number of different mechanisms including improved neuronal synaptic function in the hippocampus, alterations in calcium trafficking and suppression of inflammatory cytokines.[5–12] These data support the biological plausibility of a direct neuroprotective effect of vitamin D. However, data on the effects of supplementation of vitamin D on neurocognitive function in older adults are lacking. One small study suggested a beneficial effect of vitamin D supplementation on cognitive function,[35] while a larger study in the Women’s Health Initiative did not find any beneficial effect.[20] Further studies assessing the impact of vitamin D supplementation on neurocognitive function in populations at highest risk of cognitive decline are needed.

FGF23 is a hormone that regulates phosphorus homeostasis in part by inhibiting the activation of vitamin D. Although we hypothesized that higher FGF23 concentrations may be associated with greater risk of incident cognitive impairment via its inhibition on vitamin D activation, we found no evidence that FGF23 is associated with cognitive impairment when accounting for established risk factors. This is in agreement with a recent study in individuals with advanced chronic kidney disease that found no association of FGF23 with cognitive decline as assessed by a baseline and follow-up telephone interview screener.[36]

Our study also had limitations. We had only one baseline measure of 25(OH)D. Inclusion of only black and white adults limits our ability to extrapolate these findings to other races/ethnicities. Recent data suggest that traditional measures of vitamin D may be a poor proxy for true vitamin D status, especially among black individuals, because standard 25(OH)D assays do not discriminate between relatively inert vitamin D bound to its primary carrier protein (vitamin D binding protein) and the more biologically active free or bioavailable vitamin D.[33] We did not have 25(OH)D measurement done via liquid chromatography mass spectrometry (LC-MS), which studies suggest may be the best method to measure 25(OH)D in blood samples; nonetheless, the assay used to measure 25(OH)D in the current study has been validated against LC-MS. [37] In addition, we did not have measurements of the circulating form of Klotho, which is linked to FGF23 and may influence cognitive function [38].

In conclusion, lower 25(OH)D was associated with greater risk of incident cognitive impairment in models using race-specific tertiles in black individuals but not white individuals. If confirmed in future studies, these results suggest that targeting the markedly high prevalence of low 25(OH)D concentrations in black individuals may be a fruitful strategy for addressing racial disparities in neurocognitive outcomes in older adults.
